# Chemical Composition, Antimicrobial and Insecticidal Activities of Essential Oils of Discarded Perfume Lemon and Leaves (*Citrus Limon* (L.) Burm. F.) as Possible Sources of Functional Botanical Agents

**DOI:** 10.3389/fchem.2021.679116

**Published:** 2021-05-24

**Authors:** Panpan Wu, Xiaowen Tang, Rongchao Jian, Jiahao Li, Maoyu Lin, Huachao Dai, Kangpeng Wang, Zhaojun Sheng, Baizhong Chen, Xuetao Xu, Chen Li, Zhongze Lin, Qingmin Zhang, Xi Zheng, Kun Zhang, Dongli Li, Weiqian David Hong

**Affiliations:** ^1^ School of Biotechnology and Health Sciences, Wuyi University, Jiangmen, China; ^2^ International Healthcare Innovation Institute (Jiangmen), Jiangmen, China; ^3^ Guangdong Xinbaotang Biotechnology Co. Ltd., Jiangmen, China; ^4^ Department of Chemistry, University of Liverpool, Liverpool, United Kingdom

**Keywords:** chemical composition, antimicrobial, insecticidal, essential oil, perfume lemon, leaves

## Abstract

Two essential oils were isolated from discarded perfume lemon and leaves (*Citrus limon* (L.) Burm. F.) by hydro-distillation with good yield (0.044% for perfume lemon and 0.338% for leaves). Their biological activities were evaluated against five selected bacterial strains and *Aedes albopictus* (*Ae. albopictus*, Diptera: Culicidae). Chemical composition indicated that both essential oils were rich in essential phytochemicals including hydrocarbons, monoterpenes and sesquiterpene. These constituents revealed some variability among the oils displaying interesting chemotypes (*R*)-(+)-limonene (12.29–49.63%), citronellal (5.37–78.70%) and citronellol (2.98–7.18%). The biological assessments proved that the two essential oils had similar effect against bacterial (inhibition zones diameter ranging from 7.27 ± 0.06 to 10.37 ± 0.15 mm; MICs and MBCs ranging from 1.6 to 6.4 mg/mL); against *Ae. albopictus* larvae (LC_50_ ranging from 384.81 to 395.09 ppm) and adult mosquito (LD_50_ ranging from 133.059 to 218.962 μg/cm^2^); the activity of the two chemotypes ((*R*)-(+)-limonene and citronellal): larvae (LC_50_ ranging from 267.08 to 295.28 ppm), which were all presented in dose-dependent manners. Through this work, we have showcased that recycling and reusing of agriculture by-products, such as discarded perfume lemon and leaves can produce eco-friendly alternatives in bacterial disinfectants and mosquito control product.

## Introduction

Many mosquito-borne tropical and subtropical diseases, such as malaria, yellow fever, filariasis, dengue, and viral encephalitis (Rouis et al., 2013; Duguma, et al., 2020; Wilson, et al., 2020) contribute to a larger proportion of public health problems and a major economic burden within disease-endemic countries (Bhatt et al., 2013; Pavela, 2015). Left unchecked, insecticide resistance could lead to a substantial increase in mosquito-borne diseases incidence and mortality (Weill et al., 2003; Liu, 2015; Chang et al., 2017). Herein, urgent action is required to prevent the further development of resistance (Ranson and Lissenden, 2016) and to maintain the effectiveness of existing vector control interventions (Shaw and Catteruccia, 2019; Wilson et al., 2020). In addition to that, it would provide significant added value if the newly developed products can be used to control bacterial infection e.g. caused by scratching of the irritated skin or other inflammation reactions after mosquito bites (Ivory et al., 2015; Derua et al., 2019).

**FIGURE 1:**
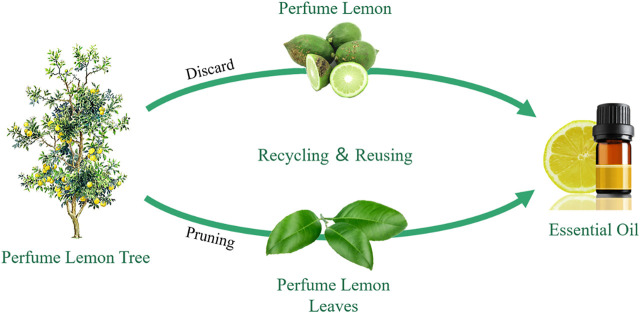
Recycling and reusing of the discarded perfume lemon and leaves.

More and more studies have been carried out and suggested that plant extractions are effective against mosquitoes at various stages of development (Bakkali et al., 2008; Gross et al., 2017). Citrus fruits (family Rutaceae) are among the most widely produced fruits all over the world (Zhang et al., 2017). The perfume lemon is a major genus of the family *Citrus limon*, which has been widely cultured in southern China. As shown in Figure 1, the discarded perfume lemon used in this study primarily resulted from commercial demand of a fixed range of fruit size, especially in the process of fruit tea preparation. Perfume lemon leaves came mainly from the pruning of perfume lemon trees, which were often discarded as a waste product, with some adverse effects on the local environment and ecology. Thus, reusing of discarded perfume lemon and leaves is not only beneficial to the comprehensive utilization of natural resources, but also can turn waste into treasure and increase the added value of the agricultural process.

The two essential oils of discarded perfume lemon and leaves were obtained by hydro-distillation; their chemical compositions were determined by gas chromatography-mass spectrometry (GC-MS) analyses (Peng et al., 2014). The aim of this study was to investigate the antibacterial and larvicidal potentials of the perfume lemon and leaf essential oils against the selected bacteria, the larval and adult *Aedes albopictus* (*Ae. albopictus*, Diptera: Culicidae). Comparison of active components and their active ingredients were also discussed. The reduced usage of synthetic insecticides by replacement of plant origin product is being considered as safe alternatives for environment and human health (Pavela, 2015; Pavela and Benelli, 2016).

## Materials and Methods

### Plant Material and Chemicals

Discarded perfume lemon and leaves (*Citrus limon* (L.) Burm. F. Appraised by Professor Junxia Zheng of Guangdong University of Technology) were obtained from the local farm (Xinghong farm, Enping, Jiangmen, Gaungdong, China). Mueller-Hinton agar (MHA) and Mueller-Hinton broth (MHB) were purchased from Guangdong Huankai Microbial Technology Co., Ltd. (Guangdong, China), custom alkanes solution of C_8_-C_40_ n-alkanes standard (Reagent Brand: o2si) was bought from ANPEL Laboratory Technologies (Shanghai, China). Deltamethrin was purchased as analytical reagent from J&K Scientific (Beijing, China). (*R*)-(+)-Limonene and citronellal were purchased from Aladdin chemical company (Shanghai, China). Other reagents were obtained as analytical reagents from Tansoole (Shanghai, China).

### Extraction and Chemical Analysis of Discarded Fresh Perfume Lemon and Leaves Essential Oils

The discarded fresh perfume lemon and leaves were suspended in distilled water with a solid-liquid ratio of 1: 4 (W/V), and the aliquot was placed in a 2.5 L round-bottomed sample flask which was then hydro-distilled for 1.5 h after the water and sample mixture started to boil. The essential oils were collected by oil-water separator, which then dried over anhydrous MgSO_4_ and filtered through a microporous membrane.

Chemical components of the two essential oils were analyzed on a Thermo Scientific TRACE 1300 Gas Chromatograph coupled to an ISQ Qd Mass Spectrometer and equipped with a TG-5 MS capillary column (30 m × 0.25 mm i.d., 0.25 μm film thickness, Thermo Scientific). Helium was used as the carrier gas with a flow rate of 1 ml/min. The oven temperature was programmed for 2 min at 60°C, then raised to 160°C at 5°C/min and held for 2 min, then increased to 260°C at 20°C/min and maintained for 20 min. The temperature of injector and MS transfer line was set to 280°C; temperature of ion source was set to 320°C. Mass spectra were recorded in the electron impact ionization (EI) at 70 eV. The scan range was from 35 to 450 m*/z.* Identification of the components of the two essential oils was based on the search results of NIST mass spectral library, together with comparisons of commercially available standards and Kovats indices (KI). The KI was determined relative to the retention times of a C_8_-C_40_ n-alkanes standard, and calculated as Eq. 1. Then the calculated KI was compared with the KI of NIST Chemistry WebBook.

$$KI=100n+\frac{100\left(t_{x}-t_{n}\right)}{t_{n+1}-t_{n}}.$$ (1)

In Eq. 1 t_x_ means the retention time of analyzed component (min); t_n_ means the retention time of n-alkane in which carbon number is n; t_n+1_ means the retention time of n-alkane in which carbon number is n+1; and t_n_＜t_x_＜t_n+1_.

### Microorganisms and Culture Media

The bacterial strains of two *Staphylococcus aureus* (ATCC 6538 and ATCC 29213), *Staphylococcus epidermidis* (ATCC 12228), *Salmonella typhimurium* (CMCC 50115) and *Escherichia coli* (CMCC 44102) were obtained from Guangdong Culture Collection Center (Guangdong, People’s Republic of China). All the five strains were cultured in Mueller-Hinton agar (MHA) and Mueller-Hinton broth (MHB).

### Agar Disk Diffusion Assay of the Two Essential Oils

The antimicrobial of essential oils were determined according to the standard agar disk diffusion method with a slight modification (Luangtongkum et al., 2007; Gaudreau et al., 2008; Benamrouche et al., 2014; Wu et al., 2018; Wu et al., 2021). A 0.5 McFarland (1 × 10^7^ to 1 × 10^8^ CFU/mL) concentration of the bacterial suspension was uniformly inoculated onto MHA solidified in 120 mm Petri dishes. Once the dishes were prepared, 6 mm-diameter discs of filter paper containing 5 μL of the examined essential oil solutions, which had been diluted with dimethyl sulfoxide (essential oil: DMSO 1:9 v/v), were pressed gently against the surface of the agar. Discs containing gatifloxacin (1 nmol) was used as positive control, while DMSO was used as the negative control. The dishes were incubated in a constant temperature incubator at 37°C for 24 h. The inhibition zone diameter was measured by a vernier caliper. All the experiments were performed in triplicate (Wu et al., 2021).

### Broth Microdilution Assay of the Two Essential Oils

The minimum inhibitory concentration (MIC) and the minimum bactericidal concentration (MBC) were determined by a microdilution method in 96-well plates according to Clinical and Laboratory Standards Institute (CLSI), with a slight modification (Sader et al., 2006; Meng et al., 2016; Wu et al., 2018; Wu et al., 2021). A series of diluted essential oils were prepared with DMSO as the solvent by two-fold serial dilution starting from a stock solution of 512 mg/mL. The final concentrations of the test essential oils were obtained in a range between 1 mg/mL and 512 mg/mL. Each well received 5 μL of a specific concentration of the essential oils and 195 μL of MHB inoculated with the test microorganism (1.5 × 10^5^ CFU/mL); the final concentrations of the examined essential oils were reached. Gatifloxacin was used as positive control and DMSO was used as negative control (Wu et al., 2021). The microplates were incubated in a bacteriological oven for 24 h at 37°C, and the susceptibility results of tested samples were monitored by measuring the absorbance at 600 nm using a Multimodel Plate Reader (Infinite 200). The lowest concentration without visible growth was defined as the MIC (Wu et al., 2021).

The minimum bactericidal concentrations (MBCs) were determined based on the MIC results (Jabrane et al., 2010; Chouaib et al., 2015; Wu et al., 2018; Wu et al., 2021): serial sub-cultivation of a 5 μL aliquots near the MIC with 195 μL fresh MHB were uniformly inoculated onto MHA solidified in 90 mm Petri dishes; incubation for 24 h at 37°C. The lowest concentration of antimicrobial agent that killed at least 99.9% of the starting inoculum was defined as the MBC endpoint, which was determined as the lowest concentration with no visible growth of bacterial colony on the Petri dishes (Wu et al., 2021). All experiments were conducted in triplicate. The final concentration of DMSO in the 96-well plate had no effect on bacterial growth.

### Killing Kinetic Studies of the Two Essential Oils

The killing kinetic studies of the two essential oils on tested bacteria strains (Theophel et al., 2014; Phee et al., 2015; Meng et al., 2016; Wu et al., 2018), including two strains of *Staphylococcus aureus* (ATCC 6538 and ATCC 29213), *Staphylococcus epidermidis* (ATCC 12228), *Salmonella typhimurium* (CMCC 50115) and *Escherichia coli* (CMCC 44102), were performed in 96-well plates, respectively. Four different concentrations (negative, 0.5 ×MIC, 1 ×MIC, 2 ×MIC) of each essential oil were studied. The microplates were incubated for 24 h at 37°C, and the growth of bacteria was monitored by measuring the absorbance at 600 nm using a Multimodel Plate Reader (Infinite 200) every 1 h (Wu et al., 2021).

### Mosquitoes

A local strain of *Ae. albopictus* larvae were collected at Huangpu District, Guangzhou, Guangdong Province, China, and had been maintained consecutively since 2013 in the laboratory. The species were reared at 14:10 light/dark photoperiod, 70 ± 5% relative humidity at 26 ± 2°C, in the laboratory of International Healthcare Innovation Institute (Jiangmen), Jiangmen, China. Larvae were fed daily with fish food, and adults were fed with 5% glucose solution. The fourth instar larvae and two to five-days-old female mosquitoes were used in this bioassay.

### Larvicidal Assay

The larvicidal activity was tested by immersion method in a 24-well plate according to the standard procedures recommended by the World Health Organization, with slight modifications (WHO, 2005; Seo et al., 2015; dos Santos Moreira et al., 2018). Firstly, the two essential oils, (*R*)-(+)-limonene and citronellal are diluted in acetone to prepare an acetone solution with a series of concentration of 0–800 ppm. Then, five larvae were transferred into the series testing solution each well for testing with no feeding during the screening. Mortality was determined after 24 h of exposure. Three replicates were carried out for each sample. The mortalities of each essential oil sample ranging from 0 to 100% were obtained. At least 10 concentrations were selected to determine the LC_20_, LC_50_, and LC_80_. Three replicates were carried out for every sample at every concentration. Deltamethrin was used as positive control while blank reference was used as negative control.

Adjusted mortality is calculated by Eq. 2:

$$\mathrm{Adjusted mortality}\left(\%\right)=\frac{\mathrm{mortality of testing group-mortality of blank group}}{1-\mathrm{mortality of blank group}}\mathrm{\times 100}.$$ (2)

### Adulticidal Bioassays

Adulticidal bioassays were assayed using Tarsal contact assay according to the reported method with slight modification (Chansang et al., 2018; Lees et al., 2019). Firstly, a graduated series of concentrations (0∼200 μg/m^2^) of deltamethrin, essential oils and the two chemotypes ((*R*)-(+)-limonene and citronellal) sample solution in acetone were prepared. Then, the sample solution was covered on the surface of 60 mm glass Petri dishes evenly. Bioassays were conducted immediately after the 4 h drying period, at 26 ± 2°C and ambient humidity. Ten female adults (2–5 days old) were exposed in each Petri dish. A 25 mL plastic deli pot with a hole melted through the base was fixed onto each Petri dish with Parafilm. Mosquitoes were introduced through the hole and, after exposing them to the treated surface for 1 h, they were aspirated out and transferred to holding cups. Then mosquitoes were put in an incubator with constant temperature (26 ± 2°C) and humidity (70 ± 5%), and fed with sugar solution. Mortality was determined after 24 h of incubation. Three duplicate trials were carried for every concentration. Blank reference was treated as negative control while deltamethrin was used as positive control. The adjusted mortality was calculated by the formula mentioned in *Larvicidal Assay* (Eq. 2). The concentration is expressed as μg of deltamethrin per m^2^ of surface of culture dishes. Regression analysis of adjusted mortalities vs. the concentrations of each examined sample to give toxicity regression equations.

## Results and Discussion

### Chemical Composition of the Two Essential Oils

**FIGURE 2:**
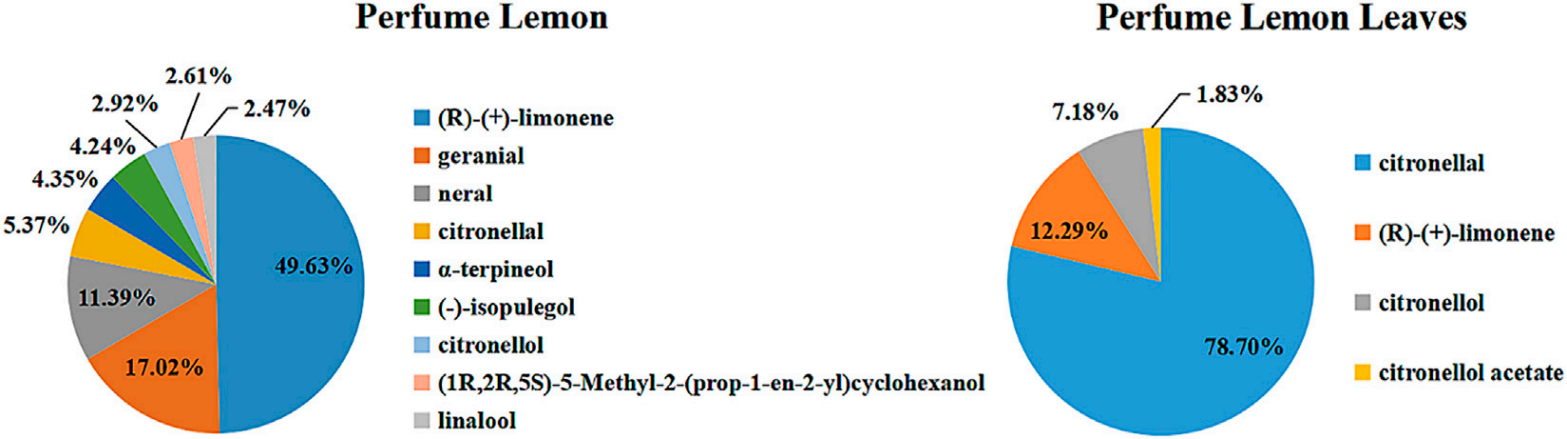
Chemical compositions of the two essential oils.

**FIGURE 3:**
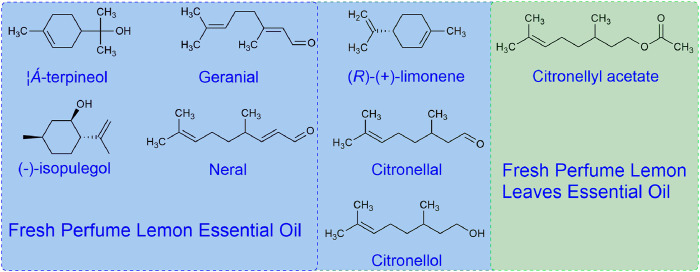
Chemical structures of major components in the two essential oils.

The essential oils extracted from discarded perfume lemon and leaves were prepared by traditional hydro-distillation with yield of 0.044% (w/w) for perfume lemon and 0.338% (w/w) for leaves. GC-MS was employed to identify the constituents and their relative content in the essential oils by the method of Kovats indices (Retention indices), and the results were as presented in Table 1 and Figure 2. The chemical structures of major components in the two essential oils were as presented in Figure 3.

**TABLE 1:** Chemical compositions of fresh perfume lemon and leaves essential oils.

No	Component^a^		RT^b^ (min)	KI^c^	KI lit^d^	Perfume lemon (%)	Perfume lemon leaves (%)
	Name	CAS no					
1	β-myrcene	123–35–3	7.546	990	990	1.49	0.56
2	octanal	124–13–0	7.871	1,002	1,002	0.49	ND
3	(R)- (+)-limonene	5,989–27–5	8.626	1,030	1,028	46.04	11.38
4	ocimene mixture of isomers	3,338–55–4	9.102	1,047	1,047	0.19	0.47
5	3,8-p-menthadiene	586–67–4	9.746	1,070	1,071	0.34	ND
6	alpha, p-dimethylstyrene	1,195–32–0	10.288	1,090	1,090	0.20	ND
7	linalool	78–70–6	10.562	1,099	1,098	2.29	0.75
8	nonanal	124–19–6	10.678	1,104	1,104	0.44	0.28
9	(−)-isopulegol	89–79–2	11.92	1,148	1,150	3.87	ND
10	citronellal	106–23–0	12.067	1,153	1,153	4.71	75.34
11	(1R,2R,5S)-5-methyl-2-(prop-1-en-2-yl) cyclohexanol	—	12.229	1,159	—	2.39	ND
12	2-(4-methyl-2,4-cyclohexadienyl)-2-propanol	1,686–20–0	12.523	1,169	1,168	1.40	ND
13	terpinen-4-ol	562–74–3	12.898	1,182	1,181	0.71	ND
14	2-(4-methylphenyl) propan-2-ol	1,197–01–9	13.03	1,187	1,187	0.43	ND
15	α-terpineol	98–55–5	13.197	1,193	1,189	4.04	ND
16	decanal	112–31–2	13.541	1,205	1,205	0.37	0.79
17	(Z)-carveol,2-methyl-5-(1-methylethenyl)-2-cyclohexen-1-ol, cis-mentha-1,8-dien-6-ol	1,197–06–4	13.658	1,209	1,210	0.19	ND
18	citronellol	106–22–9	14.18	1,229	1,228	2.70	6.68
19	neral	106–26–3	14.56	1,242	1,241	10.57	0.51
20	nerol	106–25–2	14.92	1,256	1,259	0.59	ND
21	geranial	141–27–5	15.371	1,272	1,271	15.79	0.50
22	citronellyl acetate	150–84–5	17.545	1,353	1,353	0.07	1.70
23	neryl acetate	141–12–8	18.336	1,383	1,381	0.13	0.51
24	β-caryophyllene	87–44–5	19.415	1,426	1,425	ND	0.52
25	2,6-dimethyl-6-(4-methyl-3-pentenyl)bicyclo[3.1.1]hept-2-ene	17,699–05–7	19.75	1,439	1,440	0.22	ND
26	β-bisabolene	495–61–4	21.538	1,510	1,510	0.32	ND

a Components are listed in the order of KI values. Only major components (content >0.1%) are listed in the table.

b Kovats indices (min).

c Linear Kovats indices homologous series of C_8_-C_30_ alkanes performed on a TG-5MS column.

d Linear Kovats indices were taken from https://webbook.nist.gov/chemistry/.

As indicated in Table 1 and Figure 2, 26 components were identified from the two essential oils, representing almost 100% of the total amount. The predominant component of the fresh perfume lemon essential oil was (*R*)-(+)-limonene, representing 49.63%, only 12.24% of this component was detected in the fresh leaves. In comparison, it was found that the component of citronellal in the essential oil extracted from leaves was the highest, reaching 78.35%. Other types of five alcohols and two aldehydes were detected in fresh perfume lemon essential oil, whilst 7.18% of citronellol was also found in leaves. An ester, citronellyl acetate was identified in the essential oil extracted from the leaf, but not from the fruit.

Apart from the predominant component, the lemon essential oil is composed of *d*-limonene, γ-terpinene and β-pinene, followed by four other monoterpene hydrocarbons (myrcene, sabinene, α-pinene and *p*-cimene) and other compounds in five different classes (sesquiterpene hydrocarbons, carbonyl compounds, alcohols, esters and oxides). In comparison to other previously reported analysis of essential oils from similar sources, most of the identified components are the same, but the composition of these components is different. For example, the percentages of citronellol and citronellal determined from the essential oils in this study are higher than in previous report and some of the less volatile components are also slightly different (Ciriminna et al., 2017; Yazgan et al., 2019).

### Antibacterial Activity of the Two Essential Oils

The antibacterial activity of the two essential oils was assayed against five microorganisms, including two Gram-negative bacteria strains and three Gram-positive bacteria strains. All the bacterial strains were cultured in Muller Hinton agar at 37°C overnight (Wu et al., 2021).

#### Agar Disk Diffusion Assay of the Two Essential Oils

The antimicrobial activity of the two essential oils against the five different microorganisms are summarized in Table 2. 5 μL of pure essential oil or 512 mg/mL acetone solution of essential oil on the filter paper was tested in this agar disk diffusion assay. The sizes of the inhibition zone diameters assay suggest that the antibacterial activity of the two essential oils against Gram-positive strains is higher than that against Gram-negative strains. The results also indicate that the perfume lemon essential oil was more potent than the leaves essential oil, in which the inhibition zones diameter were in the range from 7.27 ± 0.06 to 10.37 ± 0.15 mm of three tested Gram-positive *Staphylococcus* strains. However, both essential oils displayed no inhibitory activity against the two selected Gram-negative bacteria strains. Interesting, while testing the one of main components of the essential oils, (*R*)-(+)-limonene, showed similar activity against Gram-positive bacteria, this compound displayed higher antibacterial activity against Gram-negative bacteria than the essential oils.

**TABLE 2:** Biological evaluation of the two essential oils expressed in the inhibition zone (mm).

Sample code	Bacterium and inhibition zone (mm)^a^				
	Gram-positive bacteria strains			Gram-negative bacteria strains	
	*Staphylococcus aureus* (ATCC 6538)	*Staphylococcus aureus* (ATCC 29213)	*Staphylococcus epidermidis* (ATCC 12228)	*Salmonella typhimurium* (CMCC 50115)	*Escherichia coli* (CMCC 44102)
Perfume lemon essential oil (2.56 mg)	9.87 ± 1.15	8.57 ± 0.61	9.10 ± 0.78	＜6^b^	＜6
Perfume lemon essential oil (100%)^c^	10.37 ± 0.67	10.20 ± 0.52	10.37 ± 0.15	＜6	＜6
Perfume lemon leaves essential oil (2.56 mg)	8.97 ± 1.10	7.27 ± 0.06	7.53 ± 0.61	＜6	＜6
Perfume lemon leaves essential oil (100%)^c^	8.20 ± 0.26	7.37 ± 0.12	8.23 ± 0.31	＜6	＜6
(*R*)-(+)-limonene (2.56 mg)	ND^＃^	ND	ND	ND	ND
(*R*)-(+)-limonene (100%)^c^	6.77 ± 0.23	6.70 ± 0.50	7.13 ± 0.25	11.67 ± 0.32	7.93 ± 0.35
Citronellal (2.56 mg)	ND	ND	7.70 ± 0.10	ND	ND
Citronellal (100%)^c^	ND	ND	8.60 ± 0.26	ND	ND
Gatifloxacin^d^	19.12 ± 0.73	17.13 ± 0.64	18.67 ± 0.25	20.92 ± 0.72	20.78 ± 1.06

a Results are expressed as the diameter of inhibition zone (mm), values represent the means of three independent replicates ±SD.

b ＜6, no obvious inhibition zone was detected.

c Pure perfume lemon and its leaf essential oil with no dilution.

# ND, not detected.

d Gatifloxacin was used as a positive control, the dosage of gatifloxacin used in the inhibition zone assay is 1 nmol.

#### Broth Microdilution Assay of the Two Essential Oils

Using the micro-dilution method, the essential oils extracted from discarded perfume lemon and leaves were tested in the MICs and MBCs assays and the results are presented in Table 3. The similar MIC and MBC values suggested that the two essential oils had inhibitory activity through a bactericidal effect against three Gram-positive strains of *Staphylococcus*, with both MICs and MBCs ranging from 1.6 to 6.4 mg/mL. However, in contrast to the results from the agar disk diffusion assay, the results from the broth assays indicated that the essential oil extracted from the fruit was also active against the two tested strains of Gram-negative bacterial (MIC and MBC = 3.2 mg/mL), which mighty cause by some of the more volatile components, such as alcohols and aldehydes in the essential oil behaving differently in these two types of assays. Since both essential oils were derived from plant waste products, these findings indicated that recycling and reusing of these by-products can produce useful material with antibacterial activity. Moreover, it suggests that the physiochemical properties of the essential oils and their components may have further implication when apply this type of essential oils in future research and development.

**TABLE 3:** Biological Evaluation of the two essential oils expressed in MIC^a^ and MBC^b^ (mg/mL).

Sample code	MICs and MBCs of selected bacterium (mg/mL or ng/mL)									
	Gram-positive bacteria strains						Gram-negative bacteria strains			
	*Staphylococcus aureus* (ATCC 6538)		*Staphylococcus aureus* (ATCC 29213)		*Staphylococcus epidermidis* (ATCC 12228)		*Salmonella typhimurium* (CMCC 50115)		*Escherichia coli* (CMCC 44102)	
	MIC	MBC	MIC	MBC	MIC	MBC	MIC	MBC	MIC	MBC
Perfume lemon essential oil (mg/mL)	3.2	3.2	1.6	3.2	1.6	1.6	3.2	3.2	3.2	3.2
Perfume lemon leaves essential oil (mg/mL)	6.4	6.4	3.2	3.2	6.4	6.4	>12.8	>12.8	>12.8	>12.8
(*R*)-(+)-limonene (mg/mL)	4	NT^#^	512	NT	256	NT	2	NT	256	NT
Citronellal (mg/mL)	1	NT	16	NT	4	NT	128	NT	128	NT
Gatifloxacin^c^ (ng/mL)	75.08	75.08	75.08	75.08	75.08	75.08	75.08	75.08	75.08	75.08

a MICs (mg/mL), minimum inhibitory concentrations, i.e., the lowest concentration of the compound that completely inhibits the growth of bacteria.

b MBCs (mg/mL), minimum bacterial concentrations, i.e., the lowest concentration of the compound that completely kills the bacteria.

# NT, not tested.

c Gatifloxacin (ng/mL) was chosen as a positive control.

#### Killing Kinetic Studies of the Two Essential Oils

**FIGURE 4:**
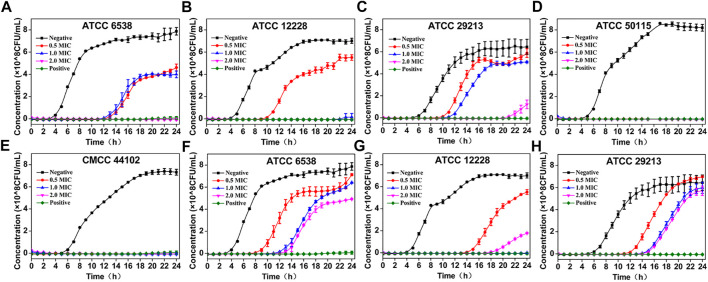
The time killing kinetic studies of the oil extracted from discarded perfume lemon and leaves against five bacteria strains. Including *Staphylococcus aureus* (ATCC 6538) **(A,F)**, *Staphylococcus epidermidis* (ATCC 12228) **(B,G)**, *Staphylococcus aureus* subsp. *Aureus* (ATCC 29213) **(C,H)**, *Salmonella typhimurium* (CMCC 50115) **(D)**, *Escherichia coli* (CMCC 44102) **(E)** and exposed to four different concentrations of the oil extracted from discarded perfume lemon (Figures 3A–E) and the oil extracted from discarded perfume leaves (Figures 3F–H) according to their respective MICs (*n* = 4), gatifloxacin was also conducted as positive control along with the two tested essential oils.

The time killing kinetic studies were assayed over a period of 24 h at 37°C according to previous study with a slightly modification (Theophel et al., 2014; Phee et al., 2015; Meng et al., 2016; Wu et al., 2018). Figure 4 displays the time-kill curves of the two essential oils against five bacteria strains, including three Gram-positive *Staphylococcus* strains (ATCC 6538, ATCC 29213, ATCC 12228), and two Gram-negative strains of *Salmonella typhimurium* (CMCC 50115) and *Escherichia coli* (CMCC 44102). As presented in Figure 4, all the bacterial strains could be effectively inhibited at the MIC of each essential oil but with a slight growth after 10 h’ examination, which may be because of their volatility during the assay. In addition, the bacterial growth could be effectively inhibited or killed at higher concentrations of 2 ×MICs and 4 ×MICs, even till the end of 24 h assay, which is also in generally accordance with the MBCs results. While the bacteria strains were incubated with the 0.5 ×MICs of both essential oils, the bacterial concentration was initially inhibited to a certain level for several hours then gradually increased, and no inhibitory effect was observed at the end of assay. Similar growth inhibition patterns were generally observed for the five selected bacteria strains in dose-dependent manner during the time killing kinetic studies of the two essential oils.

### Larvicidal Activity of the Two Essential Oils

**FIGURE 5:**
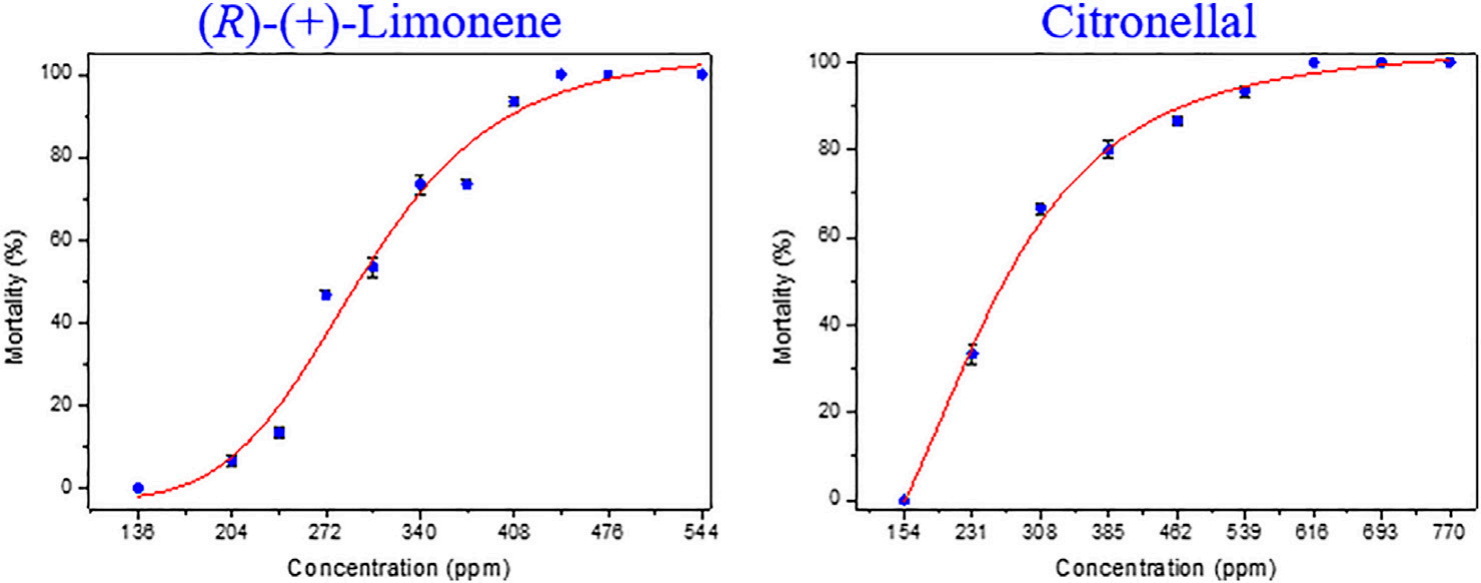
Toxicity curve of the two chemotypes against *Ae. albopictus*.

The larvicidal activities of the essential oils extracted from discarded perfume lemon and leaves and the two main components, (*R*)-(+)-limonene and citronellal in serial concentrations ranged from 0 to 650 ppm were tested against fourth instar larvae of *Ae. albopictus*. As shown in Table 4 and Figure 5, they all showed dose-dependent manner for larvicidal potency and according to the nonlinear fitting curves of the acute toxicity study of toxicity regression equations (produced by the software of Origin 8.5), LC_20_, LC_50_ and LC_80_ values were calculated from the curve fitting equations. The larvicidal activity of the oil extracted from discarded perfume lemon was LC_20_ = 248.60 ppm, LC_50_ = 384.81 ppm and LC_80_ = 595.64 ppm. The larvicidal activity of perfume lemon leaves essential oil was LC_20_ = 195.73 ppm, LC_50_ = 395.09 ppm and LC_80_ = 797.52 ppm. The larvicidal activity of the (*R*)-(+)-limonene was LC_20_ = 237.88 ppm, LC_50_ = 295.28 ppm and LC_80_ = 364.26 ppm and the activity of citronellal was LC_20_ = 198.53 ppm, LC_50_ = 267.08 ppm and LC_80_ = 382.92 ppm. The activity of these two main chemical components is comparable but slightly better than the corresponding essential oils, which may suggest that (*R*)-(+)-limonene and citronellal play a key role in the effect of essential oils on larvae.

**TABLE 4:** Acute toxicity of the two essential oils and two chemotypes against fourth instar larvae of *Ae. albopictus* (ppm).

Sample code	Toxicity regression equations	4th instar larvae of *Ae. albopictus*		
		LC_20_ (ppm)^a^	LC_50_ (ppm)^a^ (95% CI)	LC_80_ (ppm)^a^
Perfume lemon essential oil	$y=118.942-120.457/\left(1+{\left(x/384.808\right)}^{3.173}\right)$ (*R* ^2^ = 0.985)	248.60	384.81 (278.464–437.534)	595.64
Perfume lemon leaves essential oil	$y=138.471-141.968/\left(1+{\left(x/395.094\right)}^{1.974}\right)$ (*R* ^2^ = 0.970)	195.73	395.09 (204.929–383.510)	797.52
(*R*)-(+)-limonene	$y=105.266-108.298/\left(1+{\left(x/297.369\right)}^{5.864}\right)\;$(*R* ^2^ = 0.975)	237.88	295.28 (255.816–328.304)	364.26
Citronellal	$y=103.437-128.585/\left(1+{\left(x/240.245\right)}^{3.219}\right)\;$(*R* ^2^ = 0.995)	198.53	267.08 (207.284–345.576)	382.92
Deltamethrin^b^	$y=118.440-153.112/\left(1+{\left(x/0.531\right)}^{1.247}\right)$ (*R* ^2^ = 0.992)	0.175	0.531 (0.055–0.148)	1.615

a 14 serial concentrations were selected to determine LC_20_, LC_50_ and LC_80_ values. Mortality of each examined concentration was performed three replicates, five larvae each well was applied in this high throughput assay.

b Deltamethrin was treated as positive control.

### Tarsal Contact Test

**FIGURE 6:**
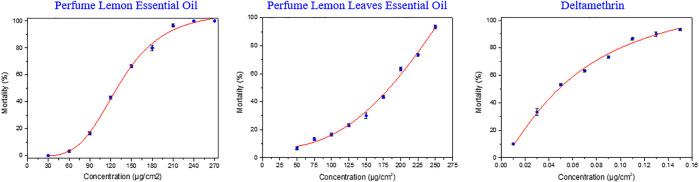
Toxicity curve of the two essential oils against *Ae. albopictus*.

A direct tarsal contact assay was performed for quantification of the anti-mosquito activity. The adulticidal bioassay of the essential oils extracted from discarded perfume lemon and leaves and the two main components (*R*)-(+)-limonene and citronellal in serial concentrations ranged from 0 to 650 μg/cm^2^ against 2–5 days old female adults of *Ae. albopictus* were performed. As shown in Table 5 and Figure 6, both essential oils revealed dose-dependent adulticidal potency and according to the nonlinear fitting curves of the acute toxicity study of toxicity regression equations (produced by the software of Origin 8.5), LD_20_, LD_50_ and LD_80_ values were calculated according to the fitting curve equations. The adulticidal activity of the essential oils extracted from discarded perfume lemon was LD_20_ = 95.180 μg/cm^2^, LD_50_ = 133.059 μg/cm^2^, LD_80_ = 186.014 μg/cm^2^. The adulticidal activity of perfume lemon leaves was LD_20_ = 145.071 μg/cm^2^, LD_50_ = 218.962 μg/cm^2^ and LD_80_ = 330.489 μg/cm^2^, which were less active than the essential oil extracted from the fruit. Surprisingly, the two components with good activity against larvae when tested alone failed to produce promising activity against any of the mosquito larvae (mortality >50% was observed only at the higher test dosage). In the present study, GC-MS data (Table 1) reveals that besides (*R*)-(+)-limonene and citronellal there are many other terpenoids and related compounds present in the oil. Thus, the activity of the two essential oils against the adult mosquito (LD_50_ = 133.059–218.962 μg/cm^2^) may be attributed to the additive or synergistic or blend effect of many/some of the constituents. This effect has previously been observed in some other essential oils, whose activity is due to the combination of the main components, but no significant activity has been found with single components alone (Papachristos et al., 2004). Overall, these two essential oils have certain insecticidal activity against adult mosquitoes.

**TABLE 5:** Acute toxicity of the two essential oils against *Ae. albopictus* (μg/cm^2^).

Sample code	Toxicity regression equations	*Ae. Albopictus* (2–5 days)		
		LD_20_ (μg/cm^2^)^a^	LD_50_ (μg/cm^2^)^a^ (95% CI)	LD_80_ (μg/cm^2^)^a^
Perfume lemon essential oil	$y=107.424-107.878/\left(1+{\left(x/133.059\right)}^{4.138}\right)$ (*R* ^2^ = 0.996)	95.180	133.059 (106.631–142.248)	186.014
Perfume lemon leaves essential oil	$y=140.278-136.307/\left(1+{\left(x/218.962\right)}^{3.367}\right)$ (*R* ^2^ = 0.985)	145.071	218.962 (140.992–202.215)	330.489
Deltamethrin^b^	$y=127.489-128.998/\left(1+{\left(x/0.0659\right)}^{1.292}\right)$ (*R* ^2^ = 0.996)	0.0225	0.0659 (0.027–0.059)	0.193

a At least nine serial concentrations were selected to determine LD_20_, LD_50_ and LD_80_ values. Mortality of each examined concentration was performed three replicates, 10 adult female mosquitoes each concentration was applied in this assay.

b Deltamethrin was treated as positive control.

## Conclusion

In summary, discarded perfume lemon and leaves, which were often disposed as waste by-produces, were recycled and reused during this study. The two essential oils were produced by traditional hydro-distillation with good yields. Kovats indices method was applied to identify the constituents and their relative content in the essential oils by GC-MS. 26 chemical components were identified, representing almost 100% of the total amount. The bioassay results implied that both essential oils present antibacterial activity, especially for the Gram-positive *Staphylococcus* bacterial strains. Moreover, the anti-mosquito potential of the two essential oils was also clearly demonstrated in their larvicidal and adulticidal activities. Therefore, the development of the essential oils extracted from discarded perfume lemon and leaves is not only friendly to the environment and ecology, but also can provide added value to the agricultural process.

As far as the comparison between (*R*)-(+)-Limonene and Citronellal is concerned, the insecticidal activity of the oxygenated monoterpenoids is higher than that of containing hydrocarbons. This conclusion is consistent with the previous findings (Regnault-Roger et al., 1993; Papachristos et al., 2004). However, these two chemicals showed significantly lower adulticidal activity than that of essential oils in this study. This finding suggests the insecticidal activity of the researched essential oil is not directly related to the content of its main components. In addition, the mode of action of these two essential oils from this study and from other similar studies remains to be elucidated (Sheng et al., 2020). This is one of the significant barriers for the further development of plant origin essential oils whilst chemical standardization and quality control are other challenge in the development process. The study of molecular mechanisms of the active components may potentially guide the development of more efficient new vector control or antibacterial agents. Relevant research is undergoing and the results will be reported in due course.

### Statistical Analysis

The results are expressed as the mean ± standard deviation (SD).

## Data Availability

The original contributions presented in the study are included in the article/Supplementary Material, further inquiries can be directed to the corresponding authors.
